# Understanding Is a Process

**DOI:** 10.3389/fnsys.2022.800280

**Published:** 2022-03-31

**Authors:** Leslie M. Blaha, Mitchell Abrams, Sarah A. Bibyk, Claire Bonial, Beth M. Hartzler, Christopher D. Hsu, Sangeet Khemlani, Jayde King, Robert St. Amant, J. Gregory Trafton, Rachel Wong

**Affiliations:** ^1^711^th^ Human Performance Wing, U.S. Air Force Research Laboratory, Wright-Patterson Air Force Base, OH, United States; ^2^Tufts University, Medford, MA, United States; ^3^U.S. Army Combat Capabilities Development Command, Army Research Laboratory, Adelphi, MD, United States; ^4^Link Training & Simulation, CAE USA, Arlington, TX, United States; ^5^Navy Center for Applied Research in AI, U.S. Naval Research Laboratory, Washington, DC, United States

**Keywords:** mutual understanding, common ground, behavioral measurement, human-machine teaming, human-robot interaction, natural language processing, explainable AI, mental models

## Abstract

How do we gauge understanding? Tests of understanding, such as Turing's imitation game, are numerous; yet, attempts to achieve a state of understanding are not satisfactory assessments. Intelligent agents designed to pass one test of understanding often fall short of others. Rather than approaching understanding as a system state, in this paper, we argue that understanding is a process that changes over time and experience. The only window into the process is through the lens of natural language. Usefully, failures of understanding reveal breakdowns in the process. We propose a set of natural language-based probes that can be used to map the degree of understanding a human or intelligent system has achieved through combinations of successes and failures.

## 1. Introduction

Few would argue with the claim that intelligent behavior in humans and machines depends on *understanding*. Yet, criteria for understanding are elusive. This is because, as this special issue motivates, we know little conclusively about the mechanisms, representations, learning and reasoning that comprise and demonstrate understanding; an ongoing challenge for researchers is to differentiate the unique character of understanding from other cognitive behaviors. A critical step toward establishing a unifying theoretical framework for understanding in both humans and machines is to establish common measures and metrics that elucidate the degree of understanding achieved within candidate frameworks or intelligent systems in a consistent way.

One component of this is clearly articulating what researchers should accept as evidence for understanding, including what constitutes the central tests of a system's ability to understand its input. Hannon (2021) identified a plausible set of criteria for characterizing understanding: understanding is a cognitive achievement, not gained simply by receiving information; understanding comes in degrees; understanding manifests itself through abilities or know-how, especially being able to “grasp” connections. There remains wide disagreement about these basics and even about more fundamental questions, such as whether understanding is a form of knowledge (and thus also subject to questions about the nature of knowledge). But this suggests a single system may exhibit multiple levels of understanding, and these will change over time. Accordingly, the evidence and critical tests should accommodate multiple degrees and adapt over time. Instead of treating understanding as an outcome, it may be more fruitful to consider the question: how does understanding support intelligent behaviors?

In this paper, we argue that understanding is a process, not an outcome. It depends on learning, interpreting, generalizing, and acting upon information. No single test is sufficient for demonstrating that one agent understands another. Indeed, understanding is not a singular type of knowledge (see also, Hannon, 2021). Assessing understanding requires probing the extent of understanding; that is, we need to execute a series of appropriately designed tests that probe the manner and extent to which information has been learned, interpreted, generalized and acted upon. The ability to probe, and therefore demonstrate any degree of, understanding requires natural language.

This paper is organized as follows. Section 1.1 reviews approaches to characterizing understanding from cognitive science and education. Many efforts in these areas attempted to establish comprehensive operational definitions and task-based benchmarks. We identify how agents falling short of desired task performance targets prompts a natural process of probing. Section 2 reviews the closely associated history of major challenge tests for computational intelligence, which place tests of understanding in natural language conversation contexts. Section 3 examines how the challenge of achieving natural language processing in machines has prompted different benchmarks across many levels of meaning representation; both successes and failures at each level illustrate the extent of understanding enabled by each level. Section 4.1 considers the constructive nature of conversation and how humans create mutual understanding through common ground. Despite advances in non-verbal cues for natural interactions (Section 4.2), common ground is a hard challenge for machines, particularly robots. If understanding is a process, then the current inability for machines to understand humans may stem from the inability of machines to engage in the language-dependent process of understanding. Section 5 reviews mental models and theory of mind methods for verbally eliciting knowledge and reasoning from humans. Section 6 reviews recent research on explainable artificial intelligence (XAI), illustrating how machines can make transparent their underlying operations. We synthesize these various approaches from cognitive science, education, natural language understanding, linguistics, verbal protocols, and XAI, to outline a method to craft *probes of understanding* to examine the understanding process. We argue that by establishing such probes in the context of interest, we identify what constitutes evidence for understanding. Thus, we can align the results of probing with the degree to which the desired understanding in humans and machines is achieved and systematically compare hypotheses about the mechanisms underpinning understanding.

### 1.1. Attempts to Define Understanding

Several broad definitions have been proposed in the cognitive sciences with a goal of establishing a definition that applies to both human and artificial intelligence (AI). For example, Hough and Gluck (2019) recently defined understanding as “The acquisition, organization, and appropriate use of knowledge to produce a response directed toward a goal, when that action is taken with awareness of its perceived purpose” (Hough and Gluck, 2019, p. 23). This is perhaps an updated, more general version of Simon's early definitions developed in his efforts to outline the criteria for software programs capable of understanding. Simon emphasized that understanding is “a relation among a system, one or more bodies of knowledge, and a set of tasks the system is expected to perform” (Simon, 1977, p. 1070). Simon's incorporation of the task or goal for an intelligent system is an extension of Moore and Newell's definition of understanding as a relationship between a system and its appropriate use of knowledge (Moore and Newell, 1974).

Consistently, these definitions emphasize that understanding entails the use of knowledge in pursuit of a task-related goal. Subsequently, the evidence for understanding is then considered to be the ability to successfully perform a target task.

This definition is measurable and achievable within narrowly scoped problems. Narrowly scoped problems include single problem solving tasks (e.g., Towers of Hanoi, demonstrated by the UNDERSTAND program; Simon and Hayes, 1976), or simple information recall in question and answer format (e.g., Siri or similar modern natural-language-based internet search assistants). Throughout the history of AI research, we can find many examples where accomplishing task-related goals has been used to demonstrate success in achieving machine understanding (usually with parallel human demonstrations or baselines).

There is an interesting context in which these early understanding definitions were established. Parallel to the emergence of computing and the computing analogies for cognition in the 1950s and 60s, the first efforts to standardize educational assessment were being published. The first of these, *Taxonomy of Educational Objectives* (Bloom et al., 1956), avoided the use of the term understanding; instead, it emphasized knowledge, comprehension, application, analysis, synthesis, and evaluation as increasingly complex objectives for someone to acquire, interpret, and use information and skills. Revisions and alternatives to this taxonomy replaced use of comprehension with understanding, making it the second level of educational objectives. In the revised *Taxonomy of Educational Objectives*, understanding is currently defined as: “Determining the meaning of instructional messages, including oral, written, and graphic communication” (Krathwohl, 2002, p. 215). This is quite a contrast to the task-oriented definitions in the cognitive sciences. Instead of framing understanding as the successful *use* of knowledge, understanding framed as comprehension emphasizes abilities like interpretation and explanation—abilities that are heavily dependent on natural language communication^1^.

However, both the educational taxonomic framing and the task-oriented goal framing of understanding suffer the same pitfall: both frame assessment as pass or fail. An individual is able to pass the test for that level of understanding in the taxonomy or not; an individual can correctly complete the task, or not. Consequently, this pushes the whole construct of understanding to be conceptualized as an intelligent agent's state: it can understand, or it cannot.

A problem with this perspective is that one can pass a test without actually possessing the intended knowledge or skill, giving an appearance of understanding. When apparent understanding is probed or pushed, perhaps tested in a slightly different context or manner from which the information was learned, the system fails. We see this fragility of performance often for deep neural network classifiers, as evidenced by the discovery of adversarial attacks. In some attacks, very small amounts of noise added to an image can drastically change the confidence of the classifier and switch image class labels (Goodfellow et al., 2018). Very minor changes to the inputs cause sharp increases in classifier errors, indicating that the classifier only had a fragile depth to its representation of the relationship between images and their conceptual-level class assignments. This falls far short of the understanding that developers intended such systems to have.

A danger in chasing the passing of a single test for understanding is that the definition of that test and what it takes to pass become moving targets. Researchers may never agree on a single benchmark against which to measure all claims about mechanisms of understanding. Indeed, Simon (1977) is a microcosm of the dilemma. In a single paper, he lays out at least three full definitions and seven varieties of understanding, because computer programs built to demonstrate sufficient ability for one definition were not sufficient to demonstrate another (see Bobrow and Collins, 1975, for similar examples).

To move our assessments of understanding forward, researchers need to change their perspectives on understanding: namely that understanding is a series of behaviors, not a single outcome.

### 1.2. The Process of Understanding

We propose that understanding should be conceptualized as a process. Understanding is an ongoing cognitive activity of acquiring, integrating and expressing knowledge according to the task or situation at hand. The process of understanding can amount to an individual's internal reflection on their own knowledge or abilities to accomplish a self-motivated goal; the process by which multiple individuals learn about and communicate with each other while working as a team; and the process of accomplishing team or individual goals. Engaging in the process allows agents to understand themselves, other agents, and external systems or situations. Understanding as a process means that different degrees of understanding may exist in a system, particularly as the tasks or information to be understood are increasingly complex.

Failures of understanding can illustrate breakdowns in the process of understanding. They do this by spotlighting when understanding has not completely enabled success. To determine why an agent failed to understand, failures are usually probed. That is, we find ways to ask why and how thought processes were correct and under what conditions or at what point in reasoning they were not. For example, in educational settings, if a student answers a question incorrectly, they are often asked to explain how they got to the wrong answer (or even to “show their work” to provide teachers with the same information). Cognitive scientists use confusion matrices or patterns of errors to investigate failures of task performance. Both groups try to identify the nature or source of the error, and then try to move toward a state of correcting the error. Hence, probing the failures can result in better understanding. Combined with successes, failures help to map the boundaries or depths of what is and is not understood by an intelligent agent.

## 2. Approach: Probing Failures of Understanding

Assessing understanding as a *process* requires a series of tests that probe a system's successes and failures in different dimensions of understanding. Within AI and Natural Language Processing (NLP), there is a tradition of creating evaluation benchmarks and “challenge” test sets that establish measuring posts of how a system might compare to an ideal, or human-like ability. Perhaps the most well-known of these tests is the “Turing test,” proposed by Alan Turing in 1950 to address the question, “Can machines think?” (Turing, 1950). In part due to the difficulties of defining *thinking*, Turing proposed an alternate formulation to probe whether or not machines can exhibit an observable behavior requiring thinking, namely a machine's convincing participation in “the imitation game.” In this game, there is a machine, a human participant, and an “interrogator” asking questions of the two parties and viewing written answers to the questions. The interrogator asks questions to ascertain which party is the machine and which is the human. The machine would succeed in this test if it were able to convince the interrogator that it was the human. The Turing test therefore presupposes that the ability to participate in natural conversation evidences intelligent behavior.

Turing hypothesized that a machine would be able to pass his test by the year 2000, and indeed, the Turing test moved from thought experiment to implementation within the Loebner competition starting in 1991—a more limited version of the test in which the interrogator has only 5 minutes to make a determination, and there is a limited set of topics. The first system to pass this limited Turing test selected the topic “whimsical conversation.” While fluent, one must question whether such whimsical conversation actually evidences any intelligence (Shieber, 1994). There is enduring fascination with the Turing test that has inspired both a string of philosophical criticisms of it as a litmus test for intelligence as well as alternative tests.

Linguist and philosopher John Searle continued to probe the question “Can computers think?” (Searle, 1984). He concluded that no digital computer can think or “understand” language in particular after posing the “Chinese room experiment.” In the Chinese room experiment, he drew a parallel between a person locked in a room manipulating Chinese symbols according to ordering rules (i.e., syntax), but without any knowledge of the actual meaning of these symbols (i.e., semantics), and a computer question-answering system manipulating input symbols designated as questions and returning associated symbols as answers. He concluded that a person in this situation does not “understand” Chinese, and that digital computers are *always* in the Chinese room—while they can manipulate symbols in such a way as to appear to understand language and even answer questions correctly, they have access only to symbols and syntax, but never the deeper semantics behind those symbols.

Thus, we ask whether or not such evaluations can still have value in their diagnostic ability to pinpoint successes and failures of understanding, where the illusion is broken and we can no longer say that the system functions in practice, regardless of why and how. A system that understands should be able to *articulate* its comprehension and demonstrate its understanding in one or more ways that humans can assess, similar to the ways we have humans demonstrate their comprehension. As a practical matter, this often demands that the system produce responses using natural language. Indeed, we make a strong commitment to the need for processing and responding to natural language: it is only through *natural language probes* that artificial agents can establish their understanding. In the absence of natural language assessments, it may be impossible to establish whether systems are merely symbol-manipulators.

For that reason, we focus on natural language processing as a gateway to understanding in humans and machines. In the following section, we work through attempted assessments of “understanding” in natural language communication, and begin to delineate how we might probe failures in that area to begin to establish benchmarks and metrics for evaluating understanding in a broad variety of systems and tasks.

## 3. Natural Language Understanding

William James writes, “any number of impressions, from any number of sensory sources, falling simultaneously on a mind WHICH HAS NOT YET EXPERIENCED THEM SEPARATELY, will fuse into a single undivided object for that mind... The baby, assailed by eyes, ears, nose, skin, and entrails at once, feels it all as one great blooming, buzzing confusion” (James, 1890, p. 488). Although it has since become debatable how true this is of the human infant brain, this state of blooming buzzing confusion is certainly true for the machine. Similarly, De Saussure writes:

“Psychologically our thought—apart from its expression in words—is only a shapeless and indistinct mass. Philosophers and linguists have always agreed in recognizing that without the help of signs we would be unable to make a clear-cut, consistent distinction between two ideas. Without language, thought is a vague, uncharted nebula. There are no pre-existing ideas, and nothing is distinct before the appearance of language” (De Saussure, 2011, p. 111).

Again, setting aside debates as to how true this is of human thought, machines must learn how to differentiate sensory input into meaningful bundles—separate categories of the things and events of the world. Furthermore, at least in the domain of the machine's function, they must learn to do so in a way that maps reasonably well to a human's organization of the same sensory input, such that both human and machine can act upon the world in any collaborative task. Because natural language provides a set of labels for many of the discrete categories of the world that humans are familiar with, to come to any kind of understanding between human and machine, the machine must be able to map its own categories and labels to natural language. This amounts to a shared symbolic space between humans and machines, which we propose is critical for establishing understanding and certainly for probing and interrogating a system's level and failures of understanding. It is worth emphasizing that while any shared symbolic space could accomplish this goal, we specifically argue that natural language is the best choice for serving this purpose as the symbolic language most familiar to humans. By “natural language” we are referring to any modality of natural language, in contrast to an artificial, controlled language^2^. Given the fundamental nature of this shared symbolic space to understanding, we discuss in relatively great detail the current landscape of natural language understanding and its evaluation.

### 3.1. Introduction to Natural Language Understanding

One area of Natural Language Processing (NLP) is referred to as Natural Language Understanding (NLU), a term introduced by Woods (1973), who proposed using English as a query language for a lunar sciences computational system. The motivation for using English as a query language remains relevant today to a variety of applications where NLU components are included. Natural language offers an ease of communication with computational systems, given that people already know, speak, and, as argued by Woods, think, in a natural language. NLU is a higher-order text processing goal, necessarily built upon other NLP components. McCarthy (1990), first published in 1976, proposed what he thought would be the necessary sub-components for achieving NLU:

1. A “parser” that turns English into ANL [Artificial Natural Language].
2. An “understander” that constructs the “facts” from a text in the ANL.
3. Expression of the “general information” about the world that could allow getting the answers to the questions by formal reasoning from the “facts” and the “general information.”
  - The “general information” would also contain non-sentence data structures and procedures, but the sentences would tell what goals can be achieved by running the procedures. In this way, we would get the best of the sentential and procedural representations of knowledge.
4. A “problem solver” that could answer the above questions on the basis of the “facts.”

Indeed, many NLU approaches introduce a pipeline somewhat like this, including an intermediate, computer-readable semantic representation and knowledge bases that can be used to compare the represented proposition against some real-world knowledge. It is this kind of approach that is also reflected in the discussion of semantic processing requirements by Jurafsky and Martin (2009), who indicated that basic requirements include: the truth of the proposition, unambiguous representations drawing upon a specific sense inventory for handling polysemous words and different contexts, as well as the ability to complete disambiguation tasks on the level of both the word and sentence.

### 3.2. Evaluating Natural Language Understanding

Because the broader goal of NLU is based upon the composition of a variety of lower-level NLP tasks, the question of whether or not a system can successfully “understand” natural language has largely only been addressed first with respect to the particular NLP task at hand (e.g., question-answering), and by evaluating the success of the individual lower-level tasks. Within NLP, these lower-level tasks are most commonly evaluated in the following way:

1. Establish a test set: this is a set of test items, which must be novel items unseen by the system in any training phase. The ground truth result is known, generally by humans establishing this through “annotation” or labeling of text with a set of relevant labels and subsequently comparing annotations for discrepancies to establish an agreed upon “gold standard.”
2. Measure the system's ability to reproduce the “gold standard”: the most common evaluation metric for this in NLP is an F-score, also referred to as F-measure or F1, which is the harmonic mean of Precision (the number of true positive results divided by the number of all identified positive results) and Recall (the number of true positive results divided by the number of all samples that should have been identified as positive).

For a particular task, accepted baseline and state-of-the-art performance levels are often established through shared tasks, where somewhat different systems with different aims are evaluated on a common test set or suite of test sets. Thus, this is similar to the kind of “challenge” approach, described in Section 2, first established in the Turing test. A good example of a contemporary evaluation suite is The General Language Understanding Evaluation (GLUE) benchmark (Wang et al., 2018), which is a collection of resources for both training and evaluation of various types of NLU tasks. It is intended to be agnostic to the system type. The evaluation suite includes tasks related to sentiment, paraphrase, natural language inference, co-reference, as well as question-answering (many of the challenges present in this evaluation suite parallel the types of probes described in Section 6). Again, system performance on these tasks is often contingent upon the performance of upstream, basic NLP components such as word sense disambiguation and syntactic parsing. In this sense, evaluating and probing the failures of understanding within NLP can be broken down into evaluations of the system's ability to recognize and interpret units of “meaning” at various levels of language, described next.

### 3.3. Levels of Language Meaning and Understanding

The assumption that a broader NLU task presupposes smaller subtasks reflects assumptions about how and where meaning is encoded in natural language.

#### 3.3.1. Understanding Word Meaning

There is a linguistic tradition that assumes that meaning is compositional—the meaning of a sentence or phrase is made up of the meanings of its individual parts, or word meanings (e.g., Chomsky, 1980). Operating under this assumption, Word Sense Disambiguation (WSD) is a key task for NLU, wherein, given an electronic lexicon or dictionary of word senses, a sense must be assigned to a word in context. For example, the sense of *play* in “She plays the violin” is to perform on an instrument, while “She plays soccer” is to participate in a game. One of the primary challenges of WSD is the selection of an appropriate lexicon, as lexicons can vary greatly in their level of coverage as well as their sense “granularity”—or the number of distinct senses associated with a word. WordNet (Fellbaum, 1998) is probably the most well-known and widely used electronic database of English words with ontological structure. It represents one of the first large-scale efforts to add such structure to a dictionary-like resource. The organization of WordNet was, in part, inspired by work in psycholinguistics investigating how and what type of information is stored in the human mental lexicon (Miller, 1995). WordNet is divided firstly into syntactic categories—nouns, verbs, adjectives and adverbs—and secondly by semantic relations, including synonymy, antonymy, hyponymy (e.g., *tree* is a hypernym of *maple*), and meronymy (part-whole relations). These relations make up a complex network of associations that is both useful for computational linguistics and NLP, and also informative in situating a word's meaning with respect to others.

Although the original English WordNet has been so valuable so as to inspire WordNets in a variety of other languages (e.g., Vossen, 1997), the practical utility of WordNet for WSD tasks has been questioned, as formal evaluations have shown that WordNet's sense inventory is so fine-grained that it is difficult for both humans and systems to tell the difference between senses and apply the appropriate sense label in context. As a response to this, the OntoNotes sense groupings were developed (Pradhan et al., 2007). These can be thought of as a more coarse-grained view of WordNet senses, as these sense groupings were based on WordNet senses that were successively merged into more coarse-grained senses based on the results of measuring inter-annotator agreement (IAA) in tagging of the senses (Duffield et al., 2007). Essentially, where two annotators were consistently able to distinguish between two senses, the distinction was kept. Where annotators were not able to consistently distinguish between two senses, the senses were conflated into one sense. In this way, human IAA establishes the ceiling performance on the task. If humans cannot reliably agree upon the distinctions of an annotation schema, we certainly cannot expect a machine to be able to reproduce those distinctions of manually annotated training and/or test data reliably. Indeed, subsequent systems trained and tested on OntoNotes sense distinctions are able to achieve much better performance on the WSD task, as measured by F-scores in comparison to a human-annotated gold standard (e.g., Zhong et al., 2008). This has led to OntoNotes becoming a benchmark dataset for training and testing WSD systems.

#### 3.3.2. Understanding Sentence Meaning

Recognizing the meanings of all of the individual words in a sentence, however, does not allow a system to understand the overall meaning of a sentence. We must also enable a system's understanding of *how* meaning is composed, or the semantic relationships between the words. Although there are a variety of established theories as to how to determine and model the semantic relations of a sentence, one dominant assumption widely made in NLP can be summarized Jackendoff's Projection Principle (Jackendoff, 1990), which states that the basic scene denoted by a sentence (i.e., participant roles) derives from the argument structure of the head verb. Verbs structure the relationships between other words of the sentence by designating the “semantic role” that the word plays with respect to the main verb of the sentence. Semantic roles, also called “thematic roles,” refer to general classes of participants in a sentence and attempt to define the relation of the participant to the event (which is often expressed by the main verb). For example, in the sentence *Fred gave Maria a book, Fred* is the agent of the action, the *book* is the gift, and *Maria* the recipient. The nature of participation in an event for a particular word is often the same, regardless of the syntactic format of the sentence. For example, in *Fred gave a book to Maria, Maria* is still the recipient, even though *Maria* is syntactically now an object of a preposition instead of a direct object.

Identifying the semantic roles of the participants is part of the more general task of understanding the semantics of the event, which has certain semantic components regardless of the specific verb used. Whether a speaker talks of *giving, handing*, or *passing*, there is always a transfer of an entity from the giver to a recipient. Grouping verbs with similar semantics allows us to refer to their shared semantic components and participant types. To support a system's ability to recognize and interpret the semantics of a sentence in this way, a variety of resources have been developed wherein human annotators attempt to apply these theories of semantic roles and verb classes to large numbers of English verbs. This annotated data can be used as training and test data for automatic semantic role labeling (SRL), in which a system automatically interprets an the *who, what, where, when, how* of a particular event. SRL resources include the benchmark PropBank (Palmer et al., 2005) and FrameNet (Fillmore et al., 2002) verb lexicons and accompanying annotated corpora, which have been reproduced in a variety of languages.

#### 3.3.3. Understanding Constructional Meaning

NLP has made progress toward recognizing and understanding the meanings of individual words and how those meanings compose to form the meaning of the broader sentence they fall in. Yet, understanding the meaning of a sentence can remain elusive, because there are still other levels of meaning that come into play for a human-like understanding of language. One aspect of this is that, in practice, systems trained on resources that assume the Projection Principle fail to understand sentences where the semantics of participants does not stem from the semantics of the head verb. For example, consider the sentences “She blinked the snow off of her eyelashes,” and “We ate our way through New York City.” While likely readily understandable to you as the reader, such sentences can be confounding for systems that have been trained to interpret sentence meaning through the lens of the main verb, which is assumed to assign semantic roles to “the snow” and “New York City”. This approach leads our systems to expect and likely conclude that snow is something that can be blinked, and a path through New York City is something that can be ingested. Such creative language usages are pushed aside in many linguistic theories as peripheral phenomena of figurative language, unimportant for the broader understanding of language (e.g., Chomsky, 1995). However, the increasing availability of computer-readable corpora has demonstrated the prevalence of these and related phenomena, where the meaning of a sentence is somehow above and beyond the individual word level. In contrast to the Projection Principle, theories of Construction Grammar (e.g., Fillmore, 1988; Goldberg, 1995; Michaelis and Lambrecht, 1996) account for such phenomena. We have begun to see the rise of computational resources (such as the FrameNet “Constructicon”; Fillmore et al., 2012) supporting the recognition and interpretation of “constructions,” such as the *caused-motion* and *way-manner* constructions exemplified in the “blink” and “eat” sentences put forth for consideration above.

#### 3.3.4. Understanding Meaning in Conversational Context and Dialogue

Again, even if we add to our system's understanding an interpretation of such constructional meaning beyond the compositional meaning of words, we may be missing implicit information that arises from the broader context of a sentence, from real-world, experiential and cultural knowledge, or from the combination of these factors. This is the broader context involved in dialogue, where language is used in bi-directional communication between speakers or interlocutors. If we would like agents to both understand and potentially communicate about the world around them as another human might, communication *via* natural language dialogue is an appealing candidate. There are significant bodies of research in dialogue systems, which can in turn require computational semantic representations of natural language that attempt to capture all of the levels of meaning described earlier in this section, as well as the recognition of “speech acts,” or what someone is attempting to do with a particular utterance beyond its basic content.

Task-oriented spoken dialogue systems, the goal of which is broadly to identify a user's intents and then act upon them to satisfy that intent, have been an active area of research since the early 1990s. Broadly, the architecture of such systems includes (i) automatic speech recognition (ASR) to recognize an utterance in speech and convert this into text, (ii) an NLU component to identify the user's intent, and (iii) a dialogue manager to interact with the user and achieve the intended task (Bangalore et al., 2006). In the earliest of these systems, “understanding” was reduced to the task of detecting a keyword in a user's utterance after the user was prompted with a limited set of permitted options (Wilpon et al., 1990).

Accordingly, the semantic representation within such systems has, in the past, been predefined frames for particular subtasks (e.g., flight inquiry), with slots to be filled (e.g., destination city; Issar and Ward, 1993). In such approaches, the semantic representation was crafted for a specific application, making generalizability to new domains difficult if not impossible. Current approaches still model NLU as a combination of intent and dialogue act classification and slot tagging, but many have begun to incorporate recurrent neural networks (RNNs) and some multi-task learning for both NLU and dialogue state tracking (Chen et al., 2016; Hakkani-Tür et al., 2016), the latter of which allows the system to take advantage of information from the dialogue context to achieve improved NLU. Substantial challenges to these systems include working in domains with intents that have a large number of possible values for each slot and accommodation of out-of-vocabulary slot values (i.e., operating in a domain with a great deal of linguistic variability). Thus, a primary challenge today, as in the past, is representing the meaning of an utterance in a form that can exploit the constraints of a particular domain but also remain portable across domains and robust despite linguistic variability.

There is a long-standing tradition of research in semantic representation within NLP, AI, theoretical linguistics, and philosophy (see Schubert, 2015, for an overview). In this body of research, there are a variety of options that could be used within dialogue systems for NLU. However, for many of these representations, there are no existing automatic “parsers” (which automatically convert language into the representation), limiting their feasibility for larger-scale implementation. Two notable exceptions with a body of research on automatic parsing are combinatory categorial grammar (CCG; Steedman and Baldridge, 2011) and Abstract Meaning Representation (AMR; Banarescu et al., 2013). CCG parsers have already been incorporated in some current dialogue systems (Chai et al., 2014). Although promising, CCG parses closely mirror the input language, so systems making use of CCG parses still face the challenge of a great deal of linguistic variability that can be associated with a single intent. In contrast, AMR abstracts from surface variation; thus, AMR may offer more regular, consistent parses in comparison to CCG. AMR is currently being investigated for use in dialogue systems onboard robots used for search and navigation tasks (Bonial et al., 2019).

To engage in dialogue, an interlocutor must interpret the meaning of a speaker's utterance on at least two levels, as first suggested by Austin (1962): (i) its propositional content, and (ii) its illocutionary force, or the “speech act”—what the speaker is trying to *do* with the utterance in the conversational context. While the aforementioned semantic representations have traditionally sought to represent propositional content, speech act theory has sought to delineate and explicate the relationship between an utterance and its effects on the mental and interactional states of the conversational participants. Speech acts have been used as part of the meaning representation of task-oriented dialogue systems since the 1970s (Bruce, 1975; Cohen and Perrault, 1979; Allen and Perrault, 1980). For a summary of some of the earlier work in this area, see (Traum, 1999). Although the refinement and extension of Austin's (1962) hypothesized speech acts by Searle (1969) remains a canonical work on this topic, there have since been a number of widely used speech act taxonomies that differ from or augment this work, including an ISO standard (Bunt et al., 2012). Nevertheless, these taxonomies often have to be fine-tuned to the domain of interest to be fully useful.

The recognition that meaning representations for dialogue systems need to be expanded to combine different levels of interpretation is growing. For example, Bonial et al. (2020) present Dialogue-AMR, which augments standard AMR, representing the content of an utterance, with speech acts representing illocutionary force. O'Gorman et al. (2018) present a Multi-Sentence AMR corpus (MS-AMR) designed to capture co-reference, implicit roles, and bridging relations. Though not strictly speech acts, the interconnected approach to meaning that this corpus annotates is directly relevant for deducing illocutionary force in a dialogue context.

Although human-robot dialogue systems often leverage a similar architecture to that of the spoken dialogue systems described above, human-robot dialogue introduces the challenge of physically situated dialogue and the necessity for symbol and action grounding, which generally incorporate computer vision. Few systems are tackling all of these challenges at this point (but see Chai et al., 2017). Symbol grounding invokes an additional layer of meaning, as systems must be able to connect a linguistic symbol to a real-world object or event. This requires a challenging combination of both perception of the current environment, as well as real-world knowledge that guide expectations about how to assign sensory input into a category of things grouped under a particular word or label in a given language. In addition to symbol grounding, human-robot dialogue, like human-human dialogue, requires establishing and maintaining “common conversational ground” of the speakers, described further in Section 4.1.

Ontologies have commonly been used for storing, organizing, and deploying the real-world knowledge required for physically situated dialogue systems (as well as other intelligent agents). However, we note that mapping informal concepts into a formal language is a difficult and persistent problem, one in which relatively little progress has been seen. For an example, consider the difficulty of establishing that a machine understands how a box works (Davis, 2011). Even everyday physical concepts that are part of ordinary human conversation, such as near, far, short, friendly, trustworthy, and so forth, are difficult to formalize. A consequence, in part, is that a number of different foundational formalisms (upper ontologies) have been proposed: Basic Formal Ontology (Arp et al., 2015), General Formal Ontology (Herre et al., 2006), Cyc (Matuszek et al., 2006), and others. Despite the challenges, research continues in this area as there are few alternatives that offer any explainability. A research direction that may hold promise is the combination of the value of linguistic and ontological resources with the power of deep learning (e.g., Faruqui et al., 2015).

Overall, the technical landscape of NLU underscores the need for evaluating understanding as a process in which failures can arise at various stages. Probing the success of increasingly complex language understanding tasks allows us to pinpoint and address the limitations of a system's understanding. Although NLP has established a good model for evaluating systems using suites of benchmark, shared tasks, the evaluations of subtasks within NLP have not been cohesively united to establish clear and measurable evaluations of the most complex tasks that rely on lower levels of understanding. For example, there is little consensus on how to evaluate either “success” or understanding for dialogue systems (see Deriu et al., 2021, for a survey on this topic).

### 3.4. Generative Language Models

Many of the approaches to different aspects of NLU described thus far have been either semi or fully supervised machine learning, often drawing upon human-annotated training data and possibly some rule-based operations. Recently, NLP has seen the rise of generative language models (GLMs), which constitute a powerful unsupervised approach to various NLP tasks. GLMs produce likely next text based on a context of other text. This process has a surprising number of useful applications, one of which is answering questions about a text passage. This is an application where one may posit that at least certain questions would require understanding of the passage to answer sensibly. One of the most dominant current GLMs is the “Generative Pre-trained Transformer” or GPT. It is a deep neural network with the transformer architecture, trained on a large general text corpus, that generates text as output, given a text prompt.

In contrast with rule-based and/or ontologically-based efforts to provide some knowledge of the meaning behind symbols, recent advances in developing massive pre-trained language models, such as GPT-3 (Brown et al., 2020), have demonstrated successes on a variety of question-answering and inference tasks. GPT-3 illustrates that computers can exploit and deploy knowledge encoded in the text in such a way as to at least broaden and deepen the illusion of understanding language. In part, this success may be attributed to the fact that GPT-3 is trained on huge amounts of text. Thus, whereas the past components that we've looked at are trained on annotated data relating to one or another level of meaning, the broader meaning of entire documents may be implicitly encoded in the GPT-3's training data, giving it a relatively broad “understanding” of meaning in the context of lots and lots of full documents, which can contain a surprising amount of cultural and real-world knowledge.

Nonetheless, GPT-3 has been criticized as “understanding” nothing—criticisms reminiscent of Searle's Chinese Room. Several recent works have set out to pinpoint and classify failures. Drawing inspiration from challenge questions meant to test the strengths and weaknesses of language models like GPT-3 in particular, we suggest the following three dimensions as a starting point for creating probes of a GLM's understanding:

1. **Knowledge Source**: Is the knowledge needed to understand an input contained in information explicitly given to the system, or in the learned world knowledge implicit in the weights acquired during training, or in linguistic knowledge that the system has learned from training?
2. **Knowledge Type**: Is the knowledge needed to understand an input about concrete entities in the world, about events and timelines, or about the contents of the minds of people? Is it about general classes and schemas, or about specific things?
3. **Reasoning Required**: What reasoning abilities are required to understand the input? Can it be answered with analogical, deductive, or inductive logic? Does it require temporal reasoning, reasoning about negation, or meta-reasoning about the motivations of the interlocutor to fully understand?

A recent analysis of the successes and failures of GPT-3 on a question-answering task, involving a carefully curated set of challenge questions, demonstrates that GPT-3 is able to successfully answer questions where the Knowledge Source is explicitly given, and can even answer questions where the knowledge type involves the contents of others' minds and some limited timeline information (Summers-Stay et al., 2021). On the other hand, it is fairly clear that GPT-3 lacks the ability to synthesize and reason about the content it has seen. In particular, GPT-3 has been shown to be unable to perform very simple mathematical operations, even when related to its text prompt, such as using addition or subtraction to determine the age of a person described in a text (Gwern, 2020; Summers-Stay et al., 2021). We suggest that this demonstrates the utility of such challenge sets in probing the failures of understanding and delineating the general areas where a particular system may lack adequate understanding for a particular application or task.

## 4. Demonstrating and Maintaining Shared Understanding

We now shift from considering natural language understanding, which can be thought of as a largely unidirectional process by which a system interprets and acts upon incoming natural language input, to considerations of how the current level of understanding is *demonstrated* by both humans and machines, and how ongoing shared understanding is maintained. This can be thought of as a bi-directional, dynamic process that may include the initial interpretation of an input, but also the ongoing efforts to subsequently demonstrate that the initial interpretation was or was not successful and then iteratively re-establish that shared understanding is being achieved as communication proceeds.

### 4.1. Conversation and Common Ground

There is longstanding documentation of the numerous behaviors in which humans engage to cultivate understanding. This includes behaviors designed to establish and maintain what is referred to as the *common ground* (Clark and Wilkes-Gibbs, 1986; Stalnaker, 2002). Common ground is the set of shared beliefs and knowledge that speakers and addressees use to appropriately situate utterances. Information becomes part of the common ground when speakers and addressees demonstrate that they *mutually accept* both the meaning that the speaker intended to convey and that the addressee has understood that meaning. Such information is then said to be *grounded* (Clark and Schaefer, 1987, 1989). The idea that *mutual* acceptance is required for grounding is part of a larger claim that conversation is the *joint* activity of the conversational participants, achieved through tightly coupled coordination rather than dissociable actions (Clark and Wilkes-Gibbs, 1986; Clark and Schaefer, 1989; Clark, 1994).

The behaviors that qualify as good “demonstrations” of mutual acceptance are complicated and varied. A behavior that may suffice in one conversational context (e.g., small talk) may be insufficient or inappropriate in another (e.g., defusing a bomb). Grounding behavior also varies as a function of the communication medium (Clark and Brennan, 1991); certain cues for grounding in face-to-face spoken conversation, such as facial expressions or intonation, are unavailable for use in text conversation, though conversational participants can leverage other features of the text medium to ground information (e.g., Potts, 2012; Mills, 2014). In all situations, speakers and addressees must mutually establish an appropriate *grounding criterion* by which to measure whether or not their behaviors demonstrate a reasonable understanding for current purposes (Clark and Wilkes-Gibbs, 1986; Clark, 1994). In some sense, speakers and addressees do not work toward “true” understanding in conversation, but rather toward the belief that there is “sufficient” understanding.

So what are some of the ways in which speakers and addressees contribute to the process of grounding? Speakers often contribute to grounding by working to prevent potential misunderstandings in the first place, such as through “self-repair” of their own utterances; for example, “He called them ‘pants’ but he meant trousers, like he used the Australian–*the American* word for trousers” where the incorrect “Australian” is immediately corrected to “American” (Schegloff et al., 1977; Clark, 1994). Speakers have been argued to prefer to repair their own utterances, and furthermore initiate those repairs themselves, rather than have their addressee indicate the need for a repair or have the addressee attempt the repair (Schegloff et al., 1977). When prevention of a production error is not possible, speakers may instead warn of possible upcoming understanding difficulties for their addressee through devices such as filled pauses (e.g., “uh” or “um”) or other *editing terms* (e.g., the use of “I mean” in an instance such as “We went to the bank–I mean the store”; see Levelt, 1983; Clark, 1994). Speakers cannot always form utterances perfectly, and thus may reformulate their utterances on the fly to improve the likelihood of understanding (Clark and Wilkes-Gibbs, 1986).

Addressees may contribute to grounding through something as simple as continued attention or providing “continuers” (also known as verbal back-channels, such as “mhhm” or “yeah”), or through something as involved as providing an overt indication of understanding through paraphrasing or repeating verbatim what the speaker said (Clark and Schaefer, 1987, 1989). Addressees may also initiate understanding repairs by requesting clarification from the speaker in a form tailored to the nature of their perceived non-understanding (Gonsior et al., 2010). It is through this collaborative effort that conversational participants achieve not only understanding but also the awareness of each other's mutual knowledge required for future conversation.

The legwork that speakers and addressees put into minimizing their *collaborative effort* (even if these contributions sometimes create greater individual effort) not only allow participants to coordinate on their mutual beliefs, but also to develop particular meanings and references as needed in the current task. Such meanings may not extend beyond that task or to new conversational participants (Clark and Wilkes-Gibbs, 1986; Brennan and Clark, 1996). These *conceptual pacts* (Brennan and Clark, 1996; Metzing and Brennan, 2003) and language routines (Mills, 2014) present an enormous challenge for human-machine understanding. Creating task-specific meanings (grounded within the task context) is not just served by knowing when and how to deploy collaborative conversational behaviors; arguably such meanings cannot be created without this kind of coordination and negotiation. It is unclear how this form of language innovation and adaptation can be created within human-machine teams until machines possess flexible grounding capabilities, tailored to the medium of communication between the team members.

The fact that human dialogue behaviors are designed to compensate for understanding failures (and such behaviors are arguably like “probing”) makes natural language dialogue a fruitful area in which to consider how we might design probes to assess the understanding of artificial systems. However, objectively identifying and quantifying failures of understanding in conversation still presents an enormous challenge. In the absence of overt behavior from the conversational participants themselves, detecting failures requires making assumptions about the mental states of the conversational participants (see Section 5). A distinction is sometimes made between failures of understanding where an addressee is aware of the failure (*non-understanding*) and failures where an addressee is not immediately aware (*misunderstanding*, e.g., Hirst et al., 1994; Weigand, 1999; Gonsior et al., 2010). In the case of non-understanding, addressees take immediate steps to remedy the failure, and therefore there is usually overt evidence in the conversation demonstrating the failure. Clark and Schaefer (1989), for example, identify at least four “states” of understanding in which addressees may believe themselves to be in, and which prompt different kinds of responses to correct the associated failure. The identification and quantification of non-understandings provide a path forward for how we might develop machines that can exhibit similar behaviors (see Gonsior et al., 2010, for one such example). Misunderstandings, on the other hand, must be detected at a later time either by the addressee, the original speaker, or both to be corrected. There may not be overt evidence of a failure at the time the failure occurs. Misunderstandings are ultimately corrected under the assumption that dialogue includes the process of “coming to an understanding,” not merely *having* understanding (Weigand, 1999). Further, conversation as a whole is still successful under the assumption that, while at any given moment the conversational participants may be misunderstanding each other, on average understanding is achieved across the entirety of the conversation (Weigand, 1999). The implicit assumption of not only collaboration but *cooperation* within conversation (Grice, 1975) allows humans to progressively and jointly establish understanding. There is much more to be learned about how speakers and addressees balance tolerating some misunderstanding under the assumption that understanding is being achieved on average, with the need to point out and correct misunderstandings as the conversation progresses. Machines, too, will need to emulate this balance to participate in conversation in a manner that would be perceived as both natural and efficient to a human.

### 4.2. Perceived Understanding

In some areas of interaction research (e.g., human robot interaction, human-agent interaction), most researchers do not work explicitly on understanding. Most researchers presumably think that understanding *per se* is too difficult a goal to reach during even short-term interaction, so the focus becomes on how to make the robot or agent *appear* as if it were understanding an interaction partner, norms of a situation, or context. We can label these sorts of approaches as *perceived understanding*. Importantly, measures of perceived understanding are usually quite straightforward: preferences and naturalness of the interaction are common metrics.

Most of the work on perceived understanding focuses on cues that the agent or robot can provide that signal that the interaction is progressing. For example, there has been a great deal of work that has shown that appropriate non-verbal communication (eye-gaze, beat gestures, facial expressions) are preferred and considered more natural than either random non-verbal communication or interactions without those cues. Trafton et al. (2008) showed that a robot system that was able to track a conversation non-verbally by looking at the speaker (based on a cognitive model of humans) was perceived as more natural than a system that acted more distracted. Other researchers have also shown that the amount, timing, and location of a robot's gaze can directly impact how much a person wants to interact with the robot (Mutlu et al., 2012; Admoni et al., 2013).

Researchers have also focused on proxemics—the amount of personal space that people maintain around themselves. Takayama and Pantofaru (2009), for example, showed that people became uncomfortable when a robot approached too close to them. Mumm and Mutlu (2011) showed that additional social cues (e.g., head gaze, likability of the robot) interacted with social distance as well. Beat gestures are another form of non-verbal signaling that can be used in interaction. For example, Huang and Mutlu (2013) showed that an agent that provides beat gestures while talking is perceived as more natural. Nods by agents and robots have also been shown to improve interaction and the naturalness of the system (Sidner et al., 2006; Arimoto et al., 2014).

Machines that demonstrate understanding of humans (whether they truly possess such understanding or not) still clearly represent an important benchmark toward creating machines that humans in turn feel they can understand (see Section 6 for further discussion on XAI). For humans to feel that they can probe the understanding of machines in the same manner as human conversational partners, machines must possess the propensity to engage collaboratively and cooperatively with humans in achieving understanding, rather than focusing on the unilateral direction of the machine understanding the human. One possible path toward unqualified human-machine partnership and understanding may require stepping back to better assess the foundations of most human collaborations. Once a common interest or goal has been realized, the next steps are likely to include considering the expectations and thought process of the other, and recognizing how these may differ from your own.

## 5. Approaching Understanding From Mental Models and Theory of Mind

A central part of the process of understanding a phenomenon is to build a model of it, i.e., a representation of its salient and functional components. Models may look very different from the phenomenon itself. For example, a watch serves as a model of the rotation of the earth. In cognitive science, human factors, and computer science, researchers agree that humans build models mentally to understand situations or other agents. When a set of individuals build mental models that overlap with one another, they are able to communicate efficiently and, as a consequence, carry out tasks that demonstrate shared understanding.

In this section, we will review the various mental model concepts and measurement methods, as well as theory of mind indicators of inferences about the state of other agents, and examine how each method may help provide insight on understanding in humans and AI systems.

### 5.1. Mental Model Definitions and Theory

There are multiple perspectives on the definition of mental models. Johnson-Laird (1983) defines mental models as small-scale mental simulations of the world we develop to enable reasoning about the environment around us. Gentner and Stevens (2014) adds that mental models are representations users develop of an environment, situation, or other agent. These models are developed through interaction with a system as well as the user's inferences about the situation or system behaviors. Mental models can be influenced by users' previous experiences such as their exposure to technology and similar systems (Gentner and Stevens, 2014). Most researchers and scientists agree on the ways mental models support intelligent behaviors:

“Mental models are the mechanisms whereby humans are able to generate descriptions of system purpose and form, explanations of system functioning and observed system states, and predictions of future system states” (Rouse and Morris, 1986, p. 3).

Shared mental models are similar; however, shared mental models are the common representations humans have about the functioning, states, and future states of systems. Shared mental models are usually investigated at a team level where the “system” being represented can be a system a team uses together or the “team” itself and its members (Cannon-Bowers et al., 1993; Kennedy et al., 2008; Jonker et al., 2010). Previous research suggests improved mental models and shared mental models are positively correlated with improved individual and team performance. Effective mental models have also been linked to better situational awareness of a system and improved metacognition (Salas et al., 1994; Scielzo et al., 2004).

### 5.2. Ways of Measuring Mental Models

There are various methods for mental model elicitation, and each measurement specifically addresses certain aspects within mental model theory. *Think Aloud* methods are one set of mental model elicitation techniques. This method encourages participants to verbally express their thought process about a situation or while completing a task. Participants are guided through the steps of describing their cognitive processes explicitly, often through verbal protocols such as think-out-loud challenges, prospect, and task reflection (Hoffman et al., 2018). One example of this technique is the Think Aloud Problem Solving Task. During this process participants verbally describe their thought process as they complete a task. This method helps to provide insight into how participants frame problems and the steps they take to solve an issue. As participants explain their thoughts, experimenters assess how participants conceptualize a system or issue (Hoffman et al., 2018). Task reflection is a similar technique, where experimenters probe participants post-task about their thought process for completing the task. These methods (e.g., structured interviews, self-explanation task, prediction task) primarily focus on the user's overall representation of the system, approach toward problem-solving, and task reflection/execution (Hoffman et al., 2018).

Another set of elicitation methods draw on how participants understand concepts and their relations to each other, typifying the various components and creating groups of similar factors. Examples of these methods include card sorting, pathfinder, and familiarity ratings. During card sorting and pathfinder methods, participants group similar concepts together and rate how similar each concept is with each other (Hoffman et al., 2018). This measure can help participants schematically represent their conceptualization of a system, its components, and the relationships among items. Diagramming is another mental model elicitation technique, where users can freely draw a pictorial representation of their cognitive process, system, or events (Hoffman et al., 2018). This method can help eliminate the bias of the experimenter on how the user pictorially represents their mental model arrays and may capture new relationships and spatial orientations of concepts.

### 5.3. Probing Mental Model Failures

Elicitation approaches can easily help researchers identify failures of understanding and gaps in someone's mental model of a system. While conducting these elicitation methods, scientists are able to identify where there is a gap in understanding and the nature of the individual's failed understanding, providing rich information to equip scientists to repair where the misunderstanding occurred. For instance, a novice mechanic could be asked to diagram the layout of an engine and to *Think Out Loud* the process they would take to complete an engine repair. With the assistance of a subject matter expert, scientists can easily determine whether the participant is lacking knowledge of the schematic layout of the engine or if the mechanic is still unfamiliar with the repair process.

While these methods seem to be very insightful for measuring users' representations of systems, these methods of mental model measurement may not have the ability to capture the entirety of understanding, especially when measuring a human's understanding of another human being. Previous research outlines the variability in mental models. Gentner and Stevens (2014) suggest that mental models are unstable mental representations. Additionally, mental models are often incomplete and lack firm boundaries. This is especially true when measuring one's mental model of an unceasingly evolving system. As teammates and humans continue to interact and gain more information about each other, mental models change. One teammate's mental model of their fellow team member may change as they continue to work together; experiences help team members learn more about their teammates' experiences and knowledge. Additionally, as a team faces new challenges together, new knowledge is built and then processed, changing each member's mental model of the world around them, their task, and their teammates. Mental model measurements also fail to capture attitudes and emotional relations between human and human mental models; these aspects are key and crucial to how mental models of teammates are used when completing tasks and relating with one another. We theorize that while mental model measurements may provide effective probing mechanisms for a user's understanding of a system, it may lack the robustness to comprehensively measure and capture a human's “understanding” of another human. Therefore, leading us to believe that understanding may be a bit more intricate and sophisticated than a mental model representation, especially when the subject of the mental model is complex and continually evolving.

### 5.4. Un-testable Theories in Theory of Mind

The shallowness of these representations is also evident for most measures of theory of mind (ToM), an extension of mental models in that both consider the knowledge or awareness of someone else, yet takes the additional step appreciating how that framework may differ from your own experience. This ability to recognize another's mental state as different from one's own is most commonly operationally measured through counterfactual reasoning or false belief (e.g., Sally-Anne task; Baron-Cohen et al., 1985, though ToM has been demonstrated across a host of situations), such completion of another's failed action and recognizing another's capacity to have concurrent yet conflicting desires (Beaudoin et al., 2020). This ability to hypothesize about the knowledge and intentions of another agent, whether living or synthetic, develops at an early age (Beaudoin et al., 2020) and is a valuable skill for social interactions and effective teaming. In human-human teams, ToM is considered critical to ensuring constructive planning and exchanges toward accomplishing a task, whereas the benefit in human-machine interactions is somewhat more ill-defined yet still seen as important (Benninghoff et al., 2013; Winfield, 2018).

As noted with mental models, numerous measures have been developed to evaluate an individual's capacity for ToM, yet the overwhelming majority of these are only sensitive to developmental stages and clinical populations (Beaudoin et al., 2020). Such tasks typically ask participants to adopt the perspective of a character in the story who has incomplete knowledge of the situation, then infer how that character is likely to respond. Moreover, most such tasks rely on drawings or situational schematics to describe a third-person account of a fictional scenario, similar to mental model elicitation approaches. However, imaging studies indicate such experiences fail to elicit the same neural response evident for actual social interactions, suggesting participants do not perceive these narratives in a way that accurately replicates personal interactions (Byom and Mutlu, 2013).

More interactive methods have been used, such as Meltzoff's behavioral re-enactment study (Meltzoff, 1995) which demonstrated that 18-month-olds were able to correctly interpret and complete target actions the experimenter initiated but did not finish. Though these results are compelling, paralleling the Chinese room experiment, it is impossible to conclude whether the toddlers had actually inferred the experimenter's intention, or were simply imitating an adult, behavior common for that age group (Jones, 2009). Additionally, studies involving neuro-typical adults have evaluated both observed behaviors in a communication game (Keysar et al., 2003) as well as self-reported experiences during daily activities (Bryant et al., 2013), and concluded that adults, although capable of forming a ToM, actually used the skill very rarely during real-world interactions.

In light of these findings for ToM, as well as those related to mental models outlined previously, the ability to generate any type of insight into the thoughts and perceptions of others is no doubt beneficial, both in casual and teaming environments. Indeed, the capacity to form mental models and ToM is particularly useful across a wide variety of inter-personal situations, such as supporting effective negotiations (de Weerd et al., 2017), and learning or adopting more sophisticated societal norms for ethics and morality (Leslie et al., 2006). It is important to note however that both mental models and ToM are thought to be beneficial precisely because they may help to avoid misunderstandings and failures in collaboration, yet implementation of the metrics discussed above offers little in the way of ensuring two agents have a shared understanding. Thus, members of a team, either human and synthetic, may adequately demonstrate these skills of social cognition, but this should not be viewed as a proxy for ensuring all teammates have a shared understanding.

## 6. Implied Definitions of Understanding: Explainable AI

One might plausibly think that artificial intelligence is at its core the study of systems that understand. (McDermott, 1976, p. 4) notes a temptation to assume away the challenge, however: “If a researcher tries to write an “understanding” program, it isn't because he has thought of a better way of implementing this well-understood task, but because he thinks he can come closer to writing the *first* implementation.” In the intervening half-century we have not yet seen that first implementation.

Relatively little research in AI explicitly addresses understanding in computer systems or its assessment (Thórisson et al., 2016). They propose that artificial understanding be treated no differently from human understanding, with conventional psychological tests being applied. They further propose that, in contrast to human testing, we have direct access to an AI system's internal program structures and memory, which may provide evidence for or against understanding: for example, whether a necessary perceptual discrimination is present, or whether a given capability has been learned or was pre-programmed.

Páez (2019), writing about systems that explain their own behavior, is also an exception. Páez holds that explanation should not be the goal for explainable AI (XAI) systems—rather, “a pragmatic and naturalistic account of understanding” should be the focus of the field. Such an account is currently lacking. Research in XAI offers promising hints about understanding, however, which we pursue in the remainder of this section. Our coverage of XAI, to include intelligible systems (Páez, 2019; Weld and Bansal, 2019), transparent systems (Castelvecchi, 2016), and related categories, will be selective. More comprehensive resources are Confalonieri et al. (2021)'s history, Vilone and Longo (2020)'s systematic review, and Mueller et al. (2019)'s meta-review and bibliography.

As a preliminary, note that it is common to probe a person's understanding of some phenomenon by requesting explanations, as in the verbal protocols discussed in Section 5; every schoolchild is familiar with “Explain this…” test questions. This is a form of abduction: we use the requests for explanations as probes, with responses providing evidence for or against specific forms of understanding. Now consider an XAI system, or even all XAI systems. We can translate the implemented explanations and explanatory processes into probes. Because we focus on probing for failure, we do not need to attribute understanding to these systems; rather, each failed probe is interpreted as demonstrating a lack of understanding.

By “translating” an explanation into a probe, we mean that an explanation is typically a carefully structured account that contains different kinds of information. Each is a potential type of probe. We outline major categories below. We label each category, describe representative types of probes found in the literature, and give an example template for a probe. For simplicity, assume that the target phenomenon to be explained (and implicitly, understood) is a behavior *y* of a given system, and that a probe is of the properties of some set of measurements *X* of the system or of the environment (which the system may be able to observe or change).

**Relevant information**. In a symbolic reasoning system, a discrete item of information may be relevant because it is required to make a potential inference (Buchanan and Shortliffe, 1984) or to enable a step in a plan (Fox et al., 2017; Chakraborti et al., 2020). Image classification systems process information in which sets of items may be relevant rather than individual items (e.g., edges or patches rather than pixels). A well-known non-XAI example is Pomerleau (1992)'s discovery that ALVINN, an early autonomous vehicle, had learned to use the amount of grass visible alongside the road as a surrogate for the road's curvature when it needed to turn, causing unexpected behavior in non-grassy settings. Comparable examples are now commonplace in XAI systems for deep learning (Xu et al., 2019). A simple probe might take the form, “Does *y* vary predictably with different values of *X*?” where *X* may represent different sets of measured variables.

**Relevant distinctions**. In some cases, in particular for systems that deal with non-discrete data, distinctions are needed even to define relevant features. These include distinguishing features in image classification, which may be highlighted as patches, colored overlays, saliency maps, etc. (Nourani et al., 2019; Xu et al., 2019), as briefly discussed in Section 6.1; differences between term frequencies in text information retrieval (Hearst, 1995); and threshold values or functions on continuous data (Buchanan and Shortliffe, 1984). A probe might take the form, for *X* known to be relevant information, “Does *y* vary predictably with different possible values of *X* only within a specific range of *X*? What is that range?”

**Relevant relationships**. Treating relationships as a separate category from items of information is largely arbitrary, in that the relationships themselves are information, as are properties of relationships. The distinction can be convenient for discussion, however. Relationships can be relevant in different ways. In explainable AI planning (Fox et al., 2017), different types of relationships between actions may be relevant: temporal ordering; “causal” relationships, i.e., in a causal-link planning sense (Young et al., 1994); the absence of predecessor actions needed for a given action; etc. A naïve Bayes classifier is considered highly explainable in that it explicitly identifies input variables relevant to the output classification variable (Kononenko, 2001). More generally, a Bayes network may be interpreted as a causal model, in which the existence of individual links is relevant: *X* may be the set of causes for *y*, for example. A simple probe might take the form, for different *X*, “If *X* were constant, at different possible values, would *y* vary predictably all of the time? None of the time?”

The probes above address “local” aspects of a phenomenon. Further, there is an emphasis on prediction, though predictive accuracy is not generally considered sufficient for explanation or understanding. We can also consider the more global structure and content of an explanation as evidence for understanding.

**Counterfactuals**. An account of what would happen under different conditions is important in explanation (e.g., Fox et al., 2017; Korpan and Epstein, 2018) in part because it can be evidence for understanding in terms of causation. Again, these are the central probes and explanations sought for theory of mind assessments. Some of the example probes expressed above have this flavor, e.g., “If the values of *X* were such and such, what would happen to *y*?”

**Generalizations and abstractions**. If we are interested in *y* under many different values of *X*, we can think of our goal as mapping out the policy that governs the system's behavior. A large number of individual samples may be adequate, but a more concise generalization may be possible, ideally one that applies to values of *X* and *y* not yet observed. This is a goal of ambitious work by Thórisson et al. (2016) in the area of artificial general intelligence. They directly define understanding of a phenomenon Φ as a set of models capable of predicting, explaining, recreating, and achieving goals with respect to Φ.

**Analogical cases**. Relatedly, if a phenomenon is understood in one domain, it may be possible to transfer that understanding to a new domain. For example, in robot behaviors, navigating to a given location and reaching out to grasp a target object generally depend on different control mechanisms and environment observations. Nevertheless the concept of “blockage of the path” is a generalization for some kinds of failure (St. Amant et al., 2019); each is a plausible analogy for the other.

For all of these types of probes, we require some ground truth against which we can compare a probe's output. Is a system capable of evaluating relevance, making appropriate distinctions, identifying related entities with respect to some phenomenon, in particular its own behavior? Can it extrapolate, answer “What if?” questions, explain how unlike situations actually share some underlying similarities? As we walk through a set of probes, we accumulate successes and failures, to give a better picture of the performance of a system or a human.

### 6.1. The Interpretability (or Lack Thereof) of Transformers

The success at demonstrating apparent understanding of GPT-3 and its subsequent variations of sizes and styles of transformer networks beg the question of its interpretability and explainability. Consequently, there is emerging work seeking to interpret the internal representations underlying transformers success; it is an active area in which researchers are starting to probe AI understanding and might further benefit from organizing the investigations by the systematic areas of probing outlined above.

Self-attention (Vaswani et al., 2017), the driving force behind the power of the transformer, has come out in front as an interpretable neural network due to its ability to link network weights to specific natural language tokens or pixels in an image; that is, it brings attention to what is important. This view is common in the literature (e.g., Xu et al., 2015; Martins and Astudillo, 2016; Choi et al., 2017; Li et al., 2017; Xie et al., 2017; Vig, 2019; Tang et al., 2020). To quote Li and colleagues: “Attention provides an important way to explain the workings of neural models, at least for tasks with an alignment modeled between inputs and outputs, like machine translation or summarization” (Li et al., 2017, p. 2).

In reality, displaying this interpretability is not as simple as one may be led to believe. However, we posit that attempts to display attention weight relationships for interpretability are an example of attempts to probe the transformer's understanding. Specifically, they are probing the relevant relationships. For example, Jain and Wallace (2019) performed extensive experiments across a variety of NLP tasks that aim to assess the validity of using attention weights as explanations for the network's predictions. They tested two lines of thinking. Attention weights should correlate with feature importance measures, and counterfactual attention weights should lead to corresponding changes in prediction. Their results suggest that even though these attention models consistently lead to indisputable improved performance on NLP tasks, the transparency, explainability, and interpretability of these models is questionable at best, especially when these models are deep and have complex connections.

Brunner et al. (2020) found similar results in their study of identifiability of attention weights and token embeddings. They found that attention weights are not identifiable, i.e., there are infinitely many attention distributions that can lead to the same internal representation and model output. However, they present Hidden Token Attribution, a gradient-based method to quantify information mixing and showcase its ability to investigate contextual embeddings in self-attention models. It seems hope is not lost on the interpretability of transformers. Chefer et al. (2021) recognize the difficulty in following connections of complex networks and have benchmarked their method on recent visual Transformer networks (such as ViT model), as well as on text classification problems (BERT). They have demonstrated the validity of their approach over existing explainability methods. In the world of transformers and attention, the question of understanding is still up for debate.

## 7. Discussion and Conclusion

We have outlined herein a set of natural language probe structures that can be adapted to different domains and applied to both human and AI understanding. Critical to evaluating theories about understanding, these can be defined independently of proposed theories and prior to any empirical evaluations. They provide the structure for independent evaluations. They also have the flexibility to adapt to different contexts for assessing understanding to provide a consistently measured body of evidence. Thus, consistent with Hannon (2021)'s recent argument, we can craft that set of criteria to define understanding through the various degrees and abilities (plural) enabled by the process of understanding.

We have argued here that natural language is the core method for probing understanding. We have highlighted that while there are many ways of showing understanding (e.g., performing well on a task), we are suggesting that language, because it is the most familiar symbolic system to humans, is the best, if not the only, method for probing understanding. We should highlight that by natural language we do not mean “perfect spoken language.” First, we realize that language can be extremely nuanced with voice tone, gesture, etc. Second, there are many forms of language that can convey many of the same signals—sign language, text, etc. Forms of language that can take advantage of multi-modal cues may convey understanding with more efficient communications. Thus, we are proposing that the more language-cues (e.g., spontaneous gesture, intonation) that are available, the more nuanced and better probes of understanding will be.

Additional complexity in structuring probes for elucidating understanding arises because sometimes we are probing understanding of the external world or mechanical systems, and sometimes we are probing an agent's understanding of another human or intelligent system, as well as whether teamed intelligent agents share mutual understanding. The process of understanding has a flexibility that can support reasoning and successful interpretation of all these types. Probes will need to flexibly adapt, because probes designed for one type of understanding may not elucidate another. In the present work, we have not yet outlined a way to translate the probe structures into specific experimental paradigms. There is likely not a single way to do this; it will depend on a number of factors, like whether you are probing humans or intelligent agents, whether you have spoken or strictly typed communications (or a combination of modalities), and whether the probes are only posed in conversation/communication tasks or if there are additional task completion targets or performance metrics to pair with the probes. Elaborating potential paradigms for putting the probes into practice is left for future work.

There remain some intelligent behaviors that systematic probing may still struggle to help measure or explain as the process of understanding unfolds. Consider the sudden ability to solve an insight problem (Metcalfe, 1986; Metcalfe and Wiebe, 1987). People are generally unable to articulate how they are trying to reason through or solve a problem prior to insight. After the “aha” moment however, people can explain the solution verbally. This is further evidence that understanding requires natural language expression. Not enough of the process has unfolded when the person cannot explain their understanding; the ability to articulate understanding marks achieving a depth of understanding that can be probed.

One possible critique of our proposal that natural language is the core method for probing understanding is that understanding can be demonstrated by performance. For example, if a robot observes a tennis player and learns how to hit various tennis shots, does it understand how to play tennis? In this scenario, the robot could have simply learned various cues for how to hit the ball (stimulus-response) or even how to move itself to win a point. However, we would argue that unless it could use symbolic communication—language of some sort—it does not actually understand the game of tennis (or even the shots it can make). For example, if the robot could describe why it would lob a ball over a net player, we would judge it to have a much better understanding of the game than if the robot could just perform the action at the right time. Along these lines, Baker et al. (2020) demonstrated the emergence of intelligent behaviors in reinforcement learning agents that did not have any NLP capabilities. This seems to be a counter argument to our natural language requirement. While the agents do move through several levels of sophistication in their coordinated activities, they do this with perfect internal knowledge of the states of each other and the environment. Take away any of this knowledge, and the coordination will falter. This suggests that the need to establish understanding within and between agents is the consequence of humans and most systems lacking perfect knowledge of the states of the other agents. That information must be communicated through a common symbolic system.

## Data Availability Statement

The original contributions presented in the study are included in the article, further inquiries can be directed to the corresponding author/s.

## Author Contributions

LB coordinated the paper and wrote Section 1, introduction and attempts at understanding definitions. SK contributed to the introduction and overall argument structure. MA and CB wrote the Sections 2 and 3 on natural language processing. SB and JT wrote Section 4 on common ground and perceived understanding. BH and JK wrote Section 5 on mental models and theory of mind. RS wrote Section 6 on XAI, with CH contributing Section 6.1 on transformers. RW contributed to the discussion, Section 7. All authors contributed to the development of the main hypotheses and to reviewing the manuscript. All authors contributed to the article and approved the submitted version.

## Funding

This research was sponsored by the U.S. Department of Defense.

## Conflict of Interest

The authors declare that the research was conducted in the absence of any commercial or financial relationships that could be construed as a potential conflict of interest.

## Publisher's Note

All claims expressed in this article are solely those of the authors and do not necessarily represent those of their affiliated organizations, or those of the publisher, the editors and the reviewers. Any product that may be evaluated in this article, or claim that may be made by its manufacturer, is not guaranteed or endorsed by the publisher.

## Footnotes

^1^The full list of understanding-related competencies are interpreting, exemplifying, classifying, summarizing, inferring, comparing, explaining (Krathwohl, 2002).

^2^A controlled language could certainly be used to achieve and interrogate understanding in a limited domain, but this places the cognitive burden of communication on the human and precludes efficient generalization to new domains, both of which can be problematic in dynamic and dangerous communication settings.
